# *Angelica gigas* root ameliorates ischaemic stroke-induced brain injury in mice by activating the PI3K/AKT/mTOR and MAPK pathways

**DOI:** 10.1080/13880209.2021.1928241

**Published:** 2021-06-01

**Authors:** Se-Eun Lee, Chiyeon Lim, Suin Cho

**Affiliations:** a Research Institute for Korean Medicine, Yangsan Campus of Pusan National University, Yangsan-si, Republic of Korea; b College of Medicine, Dongguk University, Ilsandong-gu, Republic of Korea; c School of Korean Medicine, Yangsan Campus of Pusan National University, Yangsan-si, Republic of Korea

**Keywords:** Middle cerebral artery occlusion, infarction, brain edoema, neuroprotection

## Abstract

**Context:**

Traditionally, the root of *Angelica gigas* Nakai (Umbelliferae), has long been used to treat ischaemic diseases and is considered safe in humans.

**Objective:**

To investigate the neuroprotective effects of a methanol extract of *A. gigas* root (AGmex) on the middle cerebral artery occlusion (MCAO)-induced brain injury in mice, and the underlying mechanisms.

**Materials and methods:**

Two hours of transient MCAO (tMCAO) was induced in C57BL/6 mice (MCAO control group and AGmex groups), AGmex was administered to the AGmex group at 300-3,000 mg/kg bw at 1, 1, and 24 h before tMCAO or at 1000 mg/kg bw at 1 h before and after tMCAO. Infarction volumes, tissue staining, and western blotting were used to investigate the mechanism underlying the neuroprotective effects of AGmex.

**Results:**

The median effective dose (ED_50_) could not be measured because the AGmex treatment did not reduce the infarction volume caused by 2 h of tMCAO to within 50%; however, pre-treatment with AGmex twice at 1,000 mg/kg bw before tMCAO significantly reduced the infarction volumes. The proteins related to cell growth, differentiation, and death were upregulated by this treatment, and the major recovery mechanisms appeared to involve the attenuation of the mitochondrial function of Bcl-2/Bax and activation of the PI3K/AKT/mTOR and MAPK signalling pathways in ischaemic neurons.

**Conclusions:**

This study provides evidence supporting the use of *A. gigas* root against ischaemic stroke and suggests a novel developmental starting point for the treatment of ischaemic stroke.

## Introduction

Cerebrovascular disease (CVD) is a major cause of death worldwide, and acute ischaemic stroke is a leading cause of morbidity and mortality in modern society (Mittal and Goel 2017; Shah et al. 2017; Poustchi et al. 2021). When ischaemic stroke occurs, cerebral inflammation and cell death are induced in the affected region, and deterioration of mitochondrial functions is activated by harmful stimuli such as brain arterial ischaemia and reperfusion (Sims and Muyderman 2010).

Thrombolytic agents have been used to treat stroke with some success; however, side effects such as haemorrhage, and disadvantages such as narrow therapeutic time window, limit their use. Thus, research is being actively conducted on the prevention and treatment of ischaemic stroke (Hilbrich et al. 2007).

*Angelica gigas* Nakai (Umbelliferae), also called Korean *Angelica,* is a perennial indigenous to Asian countries, and its roots are used in traditional herbal medicine (Shin and Park 2014). *A. gigas* roots are the source of Angelicae gigantis Radix (AGR) in Korea, whereas *A. sinensis* (Oliv.) Diels roots are used in China (Ji et al. 2018). According to traditional Korean medicine, stroke is mainly caused by blood stasis, and AGR continues to be widely used by traditional medicine practitioners to treat ischaemia-related diseases (Shin and Park 2014). Pharmacologically, AGR has anticancer, antibacterial, and antioxidant activities, as well as an ameliorative effect against circulatory diseases (Joo et al. 2010; Park et al. 2012; Seong et al. 2018). Studies have indicated that AGR also has neuroprotective, anti-inflammatory, and anti-dementia effects (Lee YY et al. 2003; Yan et al. 2004; Kang et al. 2005; Choi IJ et al. 2009; Song et al. 2011; Yim et al. 2011; Yoon et al. 2011; Shin and Park 2014; Cho et al. 2015; Li L et al. 2015; Oh et al. 2015; Choi HS et al. 2016).

Therefore, in the present study, the neuroprotective potential of a methanol extract of AGR (AGmex) was assessed in C57BL/6 mice using a modified version of the commonly used Koizumi’s method (Koizumi et al. 1986) to temporarily block the middle cerebral artery. In addition, we investigated the molecular mechanisms responsible for the observed effects of AGmex.

## Materials and methods

### Reagents

Phosphate-buffered saline (PBS) was purchased from Bio Basic Inc. (Markham, Ontario, Canada). 2,3,5-Triphenyl-tetrazolium chloride (TTC), cresyl violet, Evans blue (EB), and propidium iodide (PI) were obtained from Sigma-Aldrich Co. (St. Louis, MO, USA). The optimal cutting temperature compound cryostat embedding medium was obtained from Thermo Fisher Scientific (Waltham, MA, USA). Primary antibodies against B-cell lymphoma 2 (Bcl-2), bcl-2-like protein 4 (Bax), phospho-phosphoinositide 3-kinase (p-PI3K), protein kinase B (PKB, AKT), phospho-extracellular signal-regulated kinase (p-ERK), ERK, phospho-c-Jun N-terminal kinase (p-JNK), JNK, phospho-p38 mitogen-activated protein kinase (p-p38), p38, and manganese superoxide dismutase (MnSOD) were purchased from Cell Signalling Technology (Danvers, MA, USA). PI3K, p-AKT, phospho-mammalian target of rapamycin (p-mTOR), sirtuin 1 (SIRT1), and β-actin were obtained from Santa Cruz Biotechnology Inc. (Dallas, TX, USA). Aquaporin 4 (AQP4) was purchased from Abcam Inc. (Milton, Cambridge, UK). The bovine serum albumin (BSA) standard and enhanced chemiluminescence (ECL) western blotting chemiluminescent substrate were purchased from Thermo Fisher Scientific.

### Preparation of the methanol extract of AGR (AGmex)

AGR was purchased from a commercial supplier (Kwangmyung-Dang, Ulsan, Korea) in April 2017 and authenticated by Dr. Cho (Department of Korean Medicine, Pusan National University). Crushed AGR (200 g) was extracted with 99% methanol at 25 °C for 2 d, filtered, concentrated using a rotary vacuum evaporator (EYELA, Tokyo, Japan), and lyophilised to obtain AGmex. Specimens of AGR (specimen no. 2017-AGR-05) and AGmex (Voucher no. 2017Ex-AGR-05) were deposited at the Plant and Extract Bank of Pusan National University School of Korean Medicine, for future reference.

The clinical dose of AGR is 10 g/60 kg bw/d for adults, which is equivalent to 26 mg/kg bw/d of AGmex for adults and 300 mg/kg bw/d for mice. Thus, AGmex was orally administered to mice at 300–3000 mg/kg bw/d.

### Analysis of AGR with high-performance liquid chromatography (HPLC)

**Figure 1.**
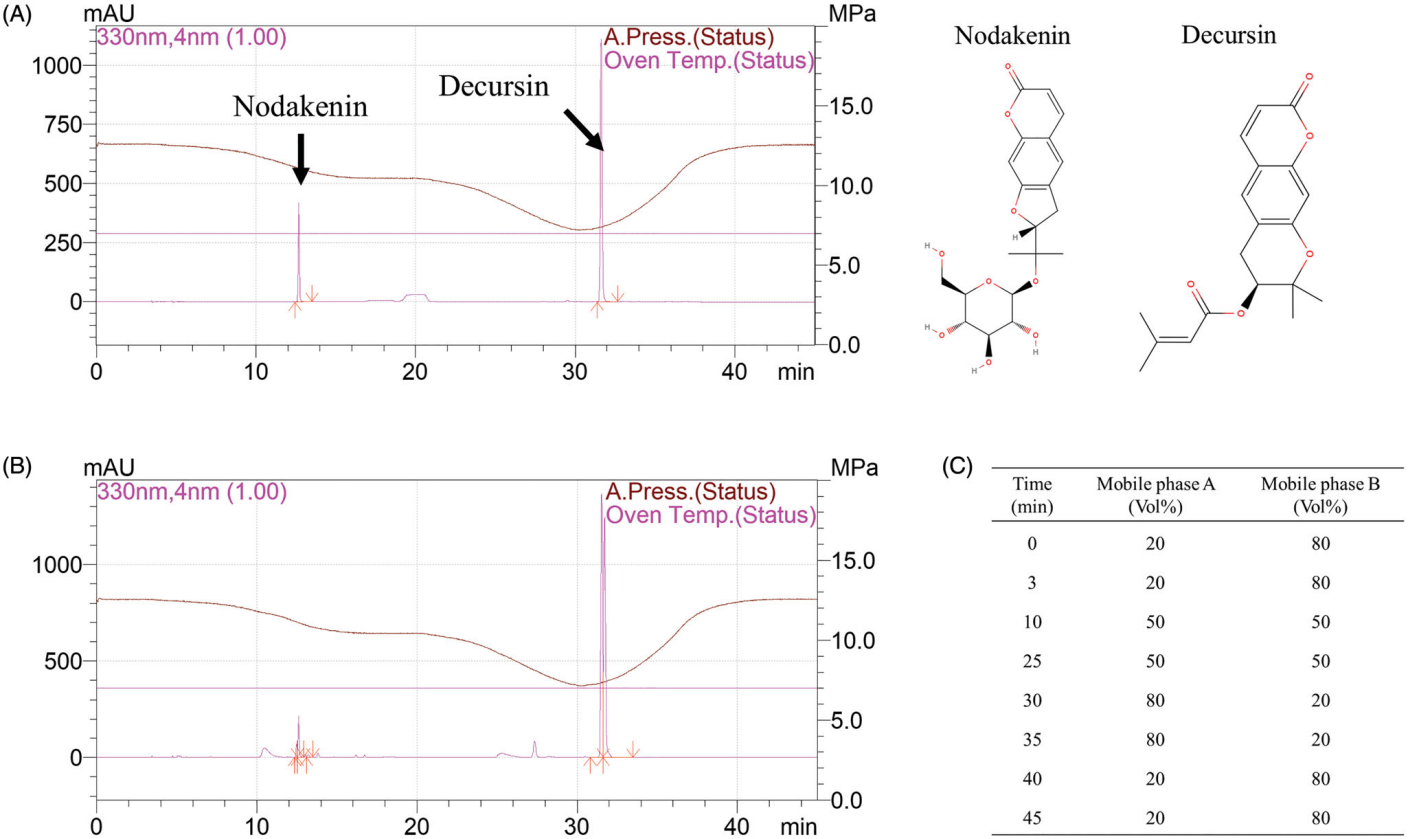
High-performance liquid chromatography (HPLC) results for the methanolic extract of *Angelica gigas* root (AGmex) and the nodakenin and decursin standards. A, HPLC chromatogram of the standards and their chemical structures. B, HPLC chromatogram of AGmex. C, Mobile phase gradient (mobile phase A, acetonitrile; mobile phase B, water). Conditions used: HPLC, Shimadzu system (Shimadzu, Kyoto, Japan); column, YMC-Triart C_18_; wavelength, 330 nm; column temperature, 35 °C; flow rate, 1 mL/min; injection volume, 10 μL.

To obtain the fingerprint of the AGmex used, we used an HPLC system (Shimadzu, Kyoto, Japan), equipped with an LC-20AD pump, an SIL-20A autosampler, an SPD-M20A detector, and a CTO-20A column oven, to determine nodakenin and decursin (the main components of AGR) levels in AGmex. The samples were separated using a YMC-Triart C_18_ column. The mobile phase and elution system are shown in Figure 1.

### Transient Middle cerebral artery occlusion (tMCAO) and AGmex administration

The most used tMCAO model is known as Koizumi’s model (Koizumi et al. 1986). In this study, we adapted Koizumi’s method to established tMCAO models, with some modifications (Lee SE et al. 2018).

**Figure 2.**
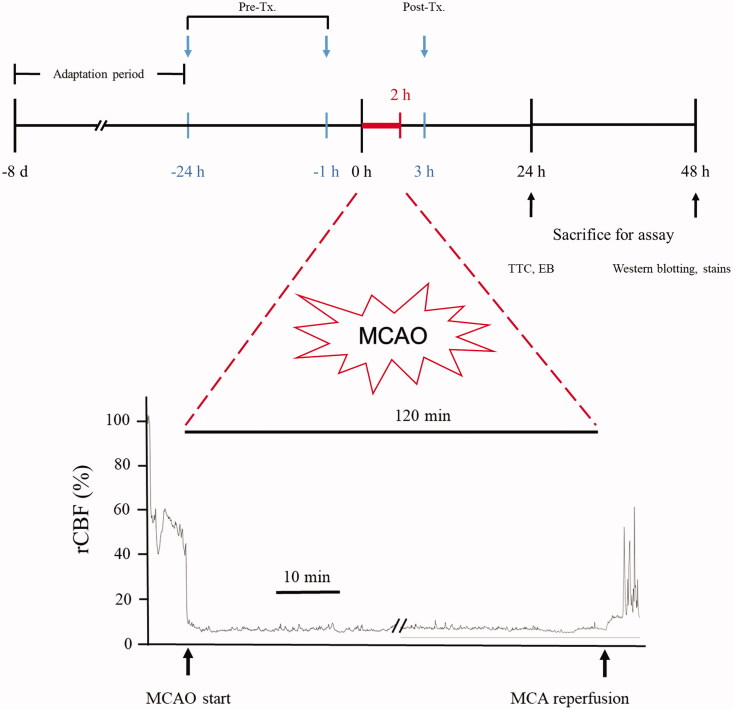
Experimental schedule of the 2 h transient middle cerebral artery occlusion (tMCAO) model. The mice were acclimated for 1 week in our animal facility before study commencement. Pre-treatment (Pre-Tx.) and post-treatment (Post-Tx.) studies were carried out to determine the timing of AGmex administration. TTC; 2,3,5-triphenyl-tetrazolium chloride; EB; Evans blue.

Mice were allocated (at least nine mice per group) to a sham-operated group; a tMCAO-operated, but not AGmex-treated group (the MCAO group); and tMCAO-operated and AGmex-treated groups, which were pre-treated with AGmex at 300, 1000, or 3000 mg/kg at 1 h (single administration) before MCAO or at 1 h and 24 h (two administrations) before MCAO, or with 1000 mg/kg of AGmex at 1 h after MCAO, respectively. Mice in the AGmex-treated groups were administered 0.5 mL of each concentration daily. Mice in the sham-operated and MCAO groups received the same amount of physiological saline as that administered with AGmex in the AGmex-treated groups. Each group consisted of at least three mice per group. Nimodipine (ND; the positive control) was also administered as a single dose of 60 mg/kg bw at 1 h post-tMCAO, as previously reported (Li JH et al. 2013; Zhao et al. 2018). A schematic of the experiment is shown in Figure 2.

In this study, researchers who performed surgery on experimental mice, researchers who conducted several assays, and a researcher who performed statistical analyses, were separately assigned. The researchers who participated in each step, then, analysed the final study results together.

### Experimental animals and induction of tMCAO

Sixty specific-pathogen-free 10-week-old C57BL/6 male mice (Samtako Bio, Osan, Korea), weighing 25–30 g, were used in this study. All procedures were approved by the Ethics Committee for Animal Care and Use at the Pusan National University (Approval No. PNU 2017-1759, certified by the Korean Association of Laboratory Animal Care). The tMCAO protocol and materials used were the same as those previously reported (Lee SE et al. 2018). During the operation, relative cerebral blood flow (rCBF) was monitored using a laser Doppler blood flow system (moorVMS-LDF; Moor Instruments, Devon, UK). The maintenance of an rCBF reduction of ≥80% for 2 h was considered to represent successful tMCAO induction. In this study, MCAO reduced the mean rCBF to 11 ± 2% (Supplementary Figure 1).

### Neurological deficit scores (NDSs) and infarct areas

NDSs were determined as described by Lee et al. (2018). In brief, the following five-point scale was adopted: 0, no neurological deficit; 1, an incomplete extension of the right forepaw; 2, no problem with voluntary movement, but turning to the right when the tail is pulled; 3, walking or circling to the right side and hypersensitivity to mechanical stimulation to its tail; 4, no response to mechanical stimulation or stroke-related death occurs. To measure infarct areas, serial 1 mm coronal sections were stained with TTC solution and digitized, and ischaemic and non-infarct tissue areas were outlined and measured using an image analysis system (Digimizer, Ostend, Belgium).

### Cardiac perfusion and brain cryosection

The mice were sacrificed by CO_2_ inhalation, and cardiac perfusion/fixation was performed using PBS containing 4% paraformaldehyde (PFA). After fixation, the brains were excised, soaked in 10% PFA, containing 10-30% sucrose, for 3 d at 4 °C, and cryosectioned at 30 μm (Leica, Wetzlar, Germany).

### Nissl and haematoxylin and eosin (H&E) staining

Brain sections on slides were dried using a slide warmer, soaked in ethanol:chloroform (1:1 vol/vol) overnight, placed in a 0.1% cresyl violet solution for 10 min, incubated for 30 min at 40 °C, washed once with distilled water, placed sequentially in 95% and 100% ethyl alcohol for 5 min each, coverslipped, sealed with mounting solution, and observed under an optical microscope (Axio; Zeiss, Oberkochen, Germany). Neuronal cell densities in the cortices were measured using the ImageJ software (NIH, MD, USA). H&E staining was performed as described previously (Lee SE et al. 2018).

### Blood-brain barrier (BBB) permeability

As the leakage of EB into the parenchyma is considered an indication of BBB disruption, BBB permeability was assessed by measuring the amount of EB, as previously described (Manaenko et al. 2011).

### Immunohistochemistry (IHC)

Sections were dried on a slide warmer, incubated with blocking serum (2% BSA) for 1 h at 25 °C, and then incubated with primary antibodies for p-mTOR and AQP4 overnight at 4 °C and with secondary antibody for 1 h at 25 °C. After washing thrice, the sections were stained with the reagent in the Envision kit (K5007; DAKO, CA, USA) for 5 min and observed under a microscope (Axio; Zeiss).

### Western blot analysis

Proteins were isolated from ipsilateral hemispheres, and lysates were obtained by centrifugation at 15,871 × g for 10 min at 4 °C. Total protein levels were determined using the BSA method. Protein samples (30 µg) were separated with SDS-PAGE (6–12%) and transferred onto PVDF membranes (Millipore, Darmstadt, Germany), which were then blocked using 5% skimmed milk, in Tris-buffered saline (TBST; 0.1% Tween 20) buffer, for 1 h at 25 °C, incubated at 4 °C overnight with primary antibodies, and incubated with secondary antibodies for 1 h at 25 °C. Membranes were then treated with ECL solution, and proteins were detected using a photosensitive luminescent analyser system (Amersham™ Imager 600; Buckinghamshire, UK). Band intensities were analysed using the ImageJ software (NIH).

### Terminal deoxynucleotidyl transferase dUTP nick end-labeling (TUNEL) staining

Sections were dried on a slide warmer, hydrated in PBS, and permeabilized for 2 min in ethanol:acetic acid (1:1 vol/vol) at 25 °C. A DeadEnd™ Fluorometric TUNEL System (Promega Corp., WI, USA) was used to visualize TUNEL-labeled nuclei, and sections were counterstained with 1 μg/mL PI to visualize all nuclei. The stained sections were observed under a fluorescence microscope (Eclipse Ts2-FL; Nikon, Tokyo, Japan).

### Statistical analysis

One-way analysis of variance (ANOVA) was used to determine the significance of differences between group mean values of the groups using Sigmaplot (version 12.0; Systat Software Inc., CA, USA). If the data did not follow a normal distribution, the Holm-Sidak test was applied for *post hoc* analysis. Results are expressed as mean ± standard deviation (SD), and differences were considered significant at *p* values < 0.05.

## Results

### Analytical comparison of AGmex with its standards

Nodakenin and decursin are the major pharmacologically active compounds found in *A. gigas.* The presence of nodakenin and decursin in AGmex was confirmed through HPLC (Figure 1(B)); the chromatograms are shown in Figure 1(A).

### Measurements of infarct volumes and edoema areas, and NDSs

**Figure 3.**
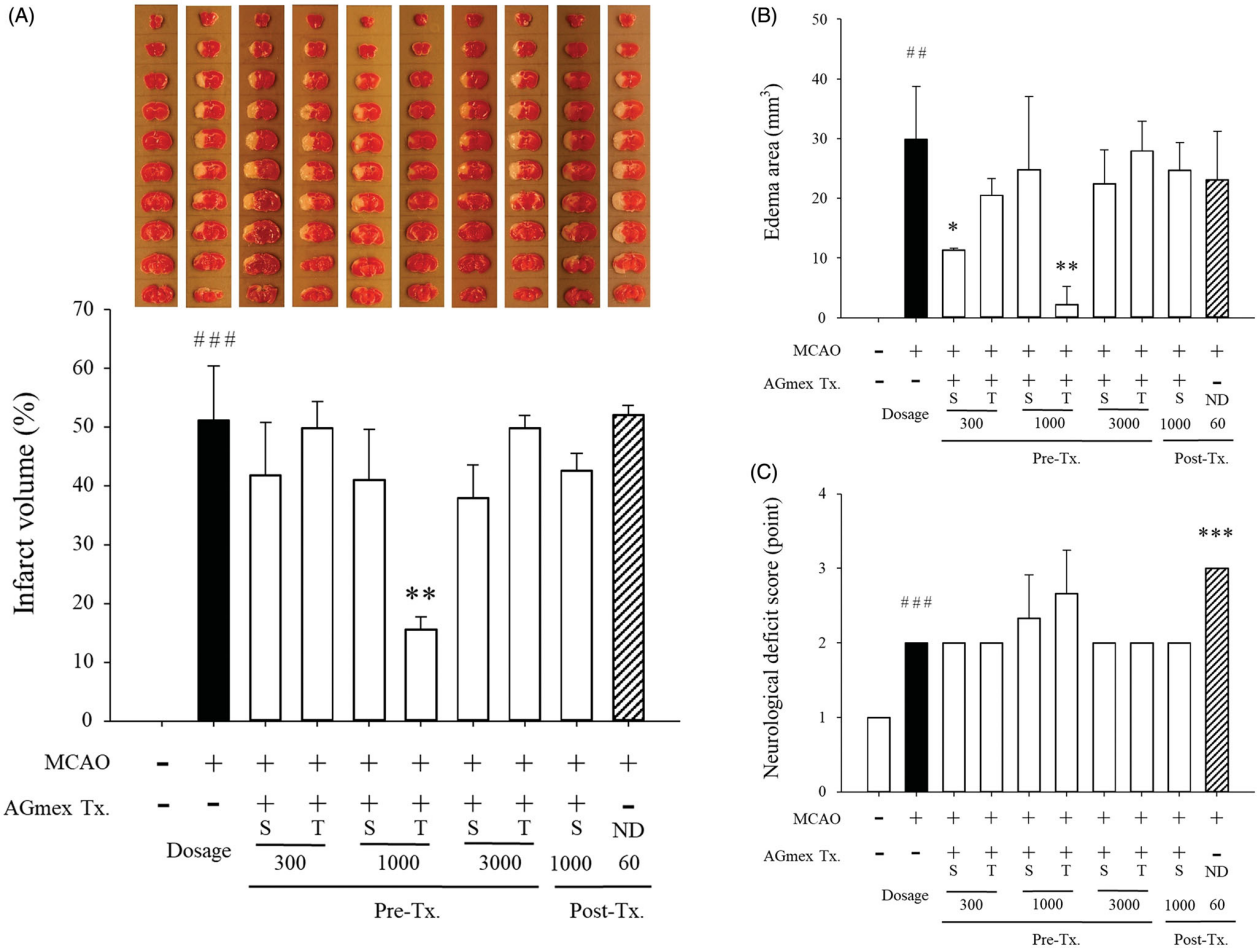
Infarct volumes, edoema areas, and neurologic deficit scores (NDS). (A) Representative photographs of TTC-stained brain coronal slices (1 mm) showing infarct areas 24 h after tMCAO (upper column) and total infarct volumes (lower column). (B) Quantitative analysis of the total edoema area. (C) Neurologic deficit score (NDS). S = single AGmex treatment at 300, 1000 or 3000 mg/kg bw p.o. and T = AGmex treatment for two consecutive days at 300, 1000 or 3000 mg/kg bw/d p.o.; ND, nimodipine (positive control), which was administered once or twice at 60 mg/kg at 1 h or 1 h and 24 h after MCAO, respectively. Results are presented as means ± SDs (*n* = 3). ##*p* < 0.01, ###*p* < 0.001 vs. the sham-operated group; *p < 0.05, **p < 0.01, ***p < 0.001 vs. the MCAO group.

When mice were pre-treated with AGmex, the most effective regimen was a double dose of 1000 mg/kg at 1 and 24 h pre-MCAO, which had a significant protective effect on infarct volume (Figure 3(A)). As 1000 mg/kg bw/d was found to be the optimal pre-treatment dose, AGmex was administered at 1000 mg/kg, 1 h after reperfusion of MCAO as a post-treatment. Although a lower percentage infarct volume was observed in the mice post-treated with 1000 mg/kg bw AGmex than in the tMCAO group, this difference was not significant. ND (positive control) was administered to mice at a dose of 60 mg/kg bw at 1 h after MCAO reperfusion but had no effect on the infarct volume (Figure 3(A)).

A significant reduction in brain edoema was observed in the mice pre-treated with AGmex once at 300 mg/kg bw or twice at 1000 mg/kg bw/day as compared with that in the tMCAO group (Figure 3(B)). No significant differences in brain edoema were observed between the tMCAO and positive control groups, and the mean NDS was significantly higher in the ND group than in the MCAO group (Figure 3(C)), suggesting that ND has an adverse effect on post-stroke behaviour. The rCBF values were similar in the tMCAO and tMCAO + AGmex groups (data not shown).

As a significant reduction in brain infarct was observed in the mice pre-treated with AGmex twice at 1000 mg/kg bw/d as compared with that in the tMCAO group, the results shown below were obtained from mice pre-treated with AGmex twice at 1000 mg/kg bw/d.

### Changes in BBB permeability

**Figure 4.**
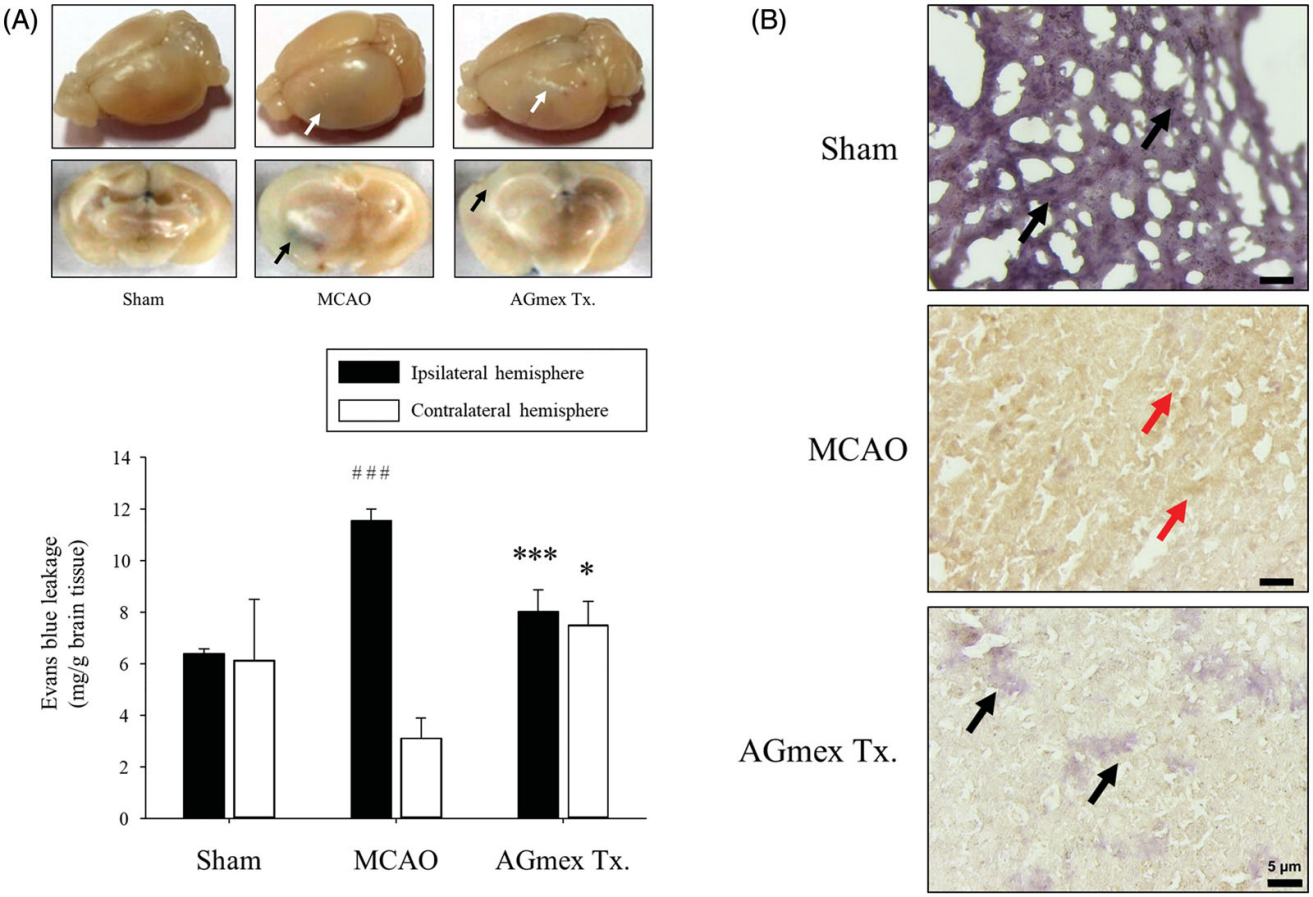
Effects of AGmex on blood-brain barrier (BBB) permeability and the localisation of AQP4 (aquaporin 4) protein in MCAO-induced mouse brains. (A) Representative images of EB extravasation in whole brain and coronal sections (upper column), and quantification of EB leakage in the ipsilateral (left) hemisphere**s** and contralateral (right) hemisphere**s** (lower column, *n* = 3). (B), Immunohistochemical (IHC) staining of AQP4 protein in the cerebral cortex (black arrows indicate negativity and red arrows positivity). Scale bars: 5 µm. Results are presented as means ± SDs (*n* = 3). ###*p* < 0.001 vs. the sham-operated group; *p < 0.05 and ***p < 0.001 vs. the MCAO group.

EB leakage in the ipsilateral hemisphere in the tMCAO group was significantly greater than that in the sham-operated group. In addition, in the AGmex 1000 mg/kg-pre-treated group, EB leakage was significantly lower than that in the tMCAO group. EB leakage was similar in the contralateral hemispheres in the sham-operated group and tMCAO group but was significantly greater in the AGmex 1000 mg/kg-pre-treated group than in the tMCAO group (Figure 4(A)).

IHC staining of ischaemic lesions in the ipsilateral hemispheres showed that AQP4 was expressed mainly in cortical neurons (red arrows; Figure 4(B)). In lesions, AQP4 expression was lower in the AGmex 1000 mg/kg-pre-treated group than in the tMCAO group (black arrows, Figure 4(B)).

### Morphological changes in neurons

**Figure 5.**
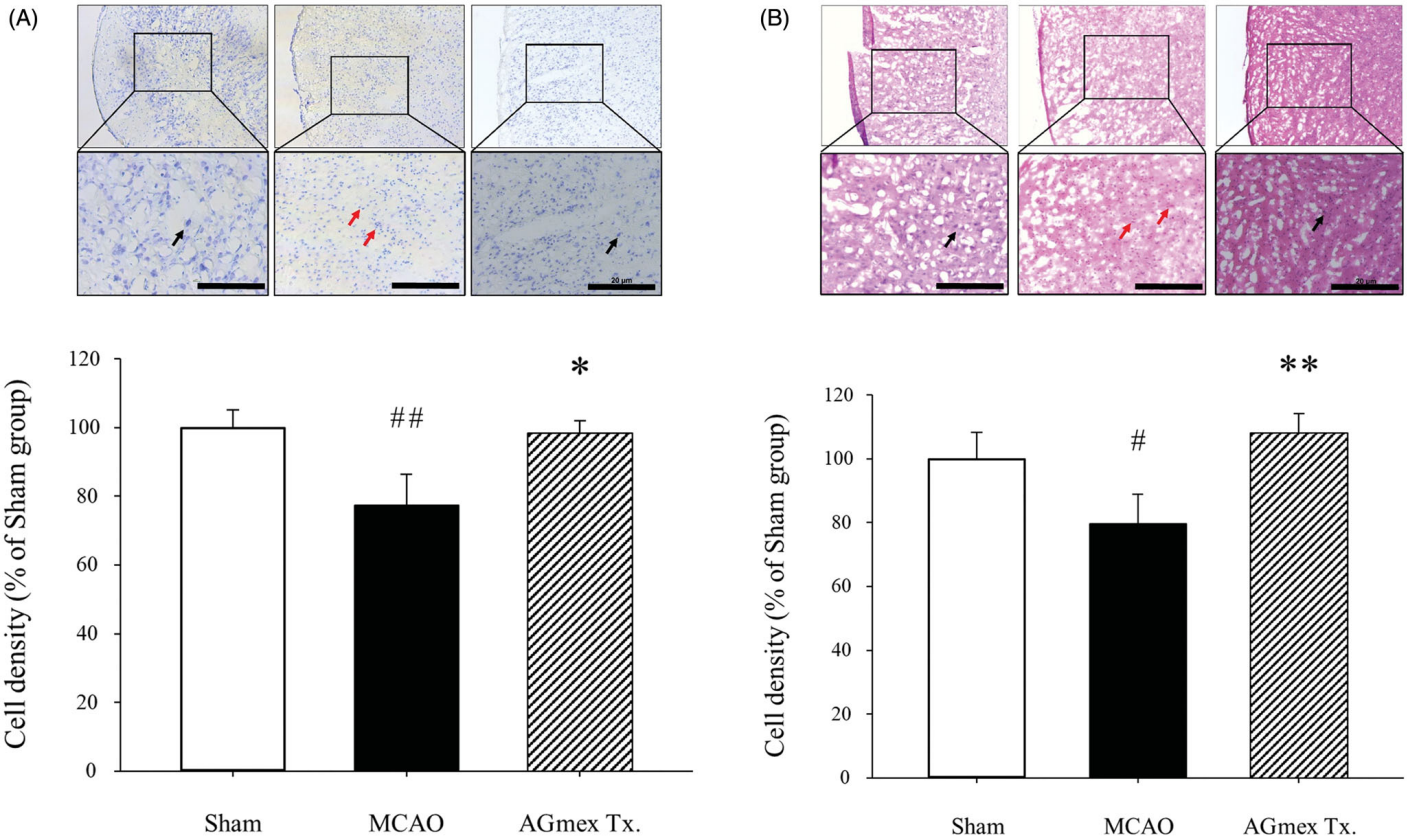
Neuroprotective effects of double pre-treatment (at 1 h and 24 h pre-MCAO) with AGmex at 1000 mg/kg on MCAO-induced cell death. (A) Representative photomicrographs of Nissl-stained neurons in the subcortical area (upper images) ischaemic ipsilateral hemisphere and results of quantitative analysis showing changes in neuron numbers following tMCAO induction and pre-treatment with AGmex (lower graph). (B) Representative photomicrographs of haematoxylin and eosin (H&E)-stained neurons (upper images) and results of quantitative analysis showing changes in neuron numbers (lower graph). Black arrows indicate intact neurons with normal morphology, and red arrows indicate neurons showing apoptotic changes and aberrant morphologies. Scale bars: 20 µm. #*p* < 0.05, ##*p* < 0.01 vs. sham controls; *p < 0.05, **p < 0.01 vs. the MCAO group.

In the sham-operated group, cresyl violet staining indicated that neurons in the tMCAO-induced subcortical region of the brain were intact, with morphologically well-arranged cytoplasm and nuclei (black arrows, Figure 5(A)). In the tMCAO group, neurons were apoptotic and showed aberrant morphologies (red arrows, Figure 5(A)). In the AGmex 1000-pre-treated group, neurons were similar to those in the sham-operated group (black arrows, Figure 5(A)).

Neurons in the subcortical brain area in the tMCAO-induced group showed DNA damage, cell shrinkage, pyknotic nuclei, and eosinophilic cytoplasm (red arrows, Figure 5(B)). However, neurons in the AGmex 1000 mg/kg-pre-treated group appeared to be much less damaged and similar to those in the sham-operated group (black arrows, Figure 5(B)).

### Apoptotic changes in ipsilateral cerebral cortices

**Figure 6.**
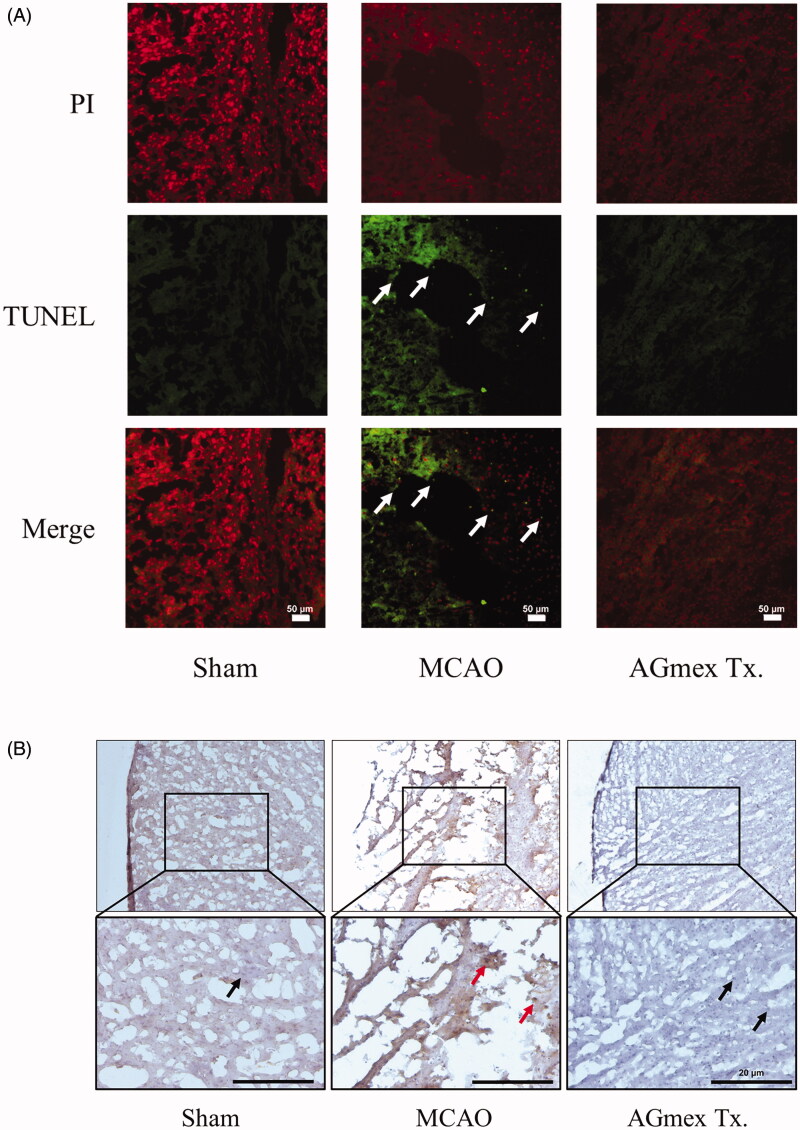
Effects of double pre-treatment (at 1 h and 24 h pre-MCAO) with AGmex at 1000 mg/kg on MCAO-induced cell death. (A) Representative images of TUNEL and PI double-stained cerebral cortex regions showing MCAO-induced apoptosis; white arrows indicate neurons showing apoptotic changes (scale bars: 50 µm). (B) Representative images of IHC staining of MCAO-induced changes in mTOR (mammalian target of rapamycin) protein expression in the cerebral subcortical region**s** of tMCAO-induced mice; red arrows indicate mTOR positivity, and black arrows indicate mTOR negativity (scale bars: 20 µm).

In the sham-operated group, neurons were PI-stained and evenly distributed, and no TUNEL-positive cells were observed. However, in the tMCAO group, TUNEL-positive neurons were obvious and highly stained and showed more condensed PI staining, indicating tMCAO-induced apoptotic changes. In contrast, PI staining showed that neurons in the AGmex 1000 mg/kg-pre-treated group exhibited nuclear condensation, but TUNEL-positive cells were not present, suggesting that AGmex had an anti-apoptotic effect (Figure 6(A)).

### Expression of cell death-related molecules in ipsilateral cerebral cortices

**Figure 7.**
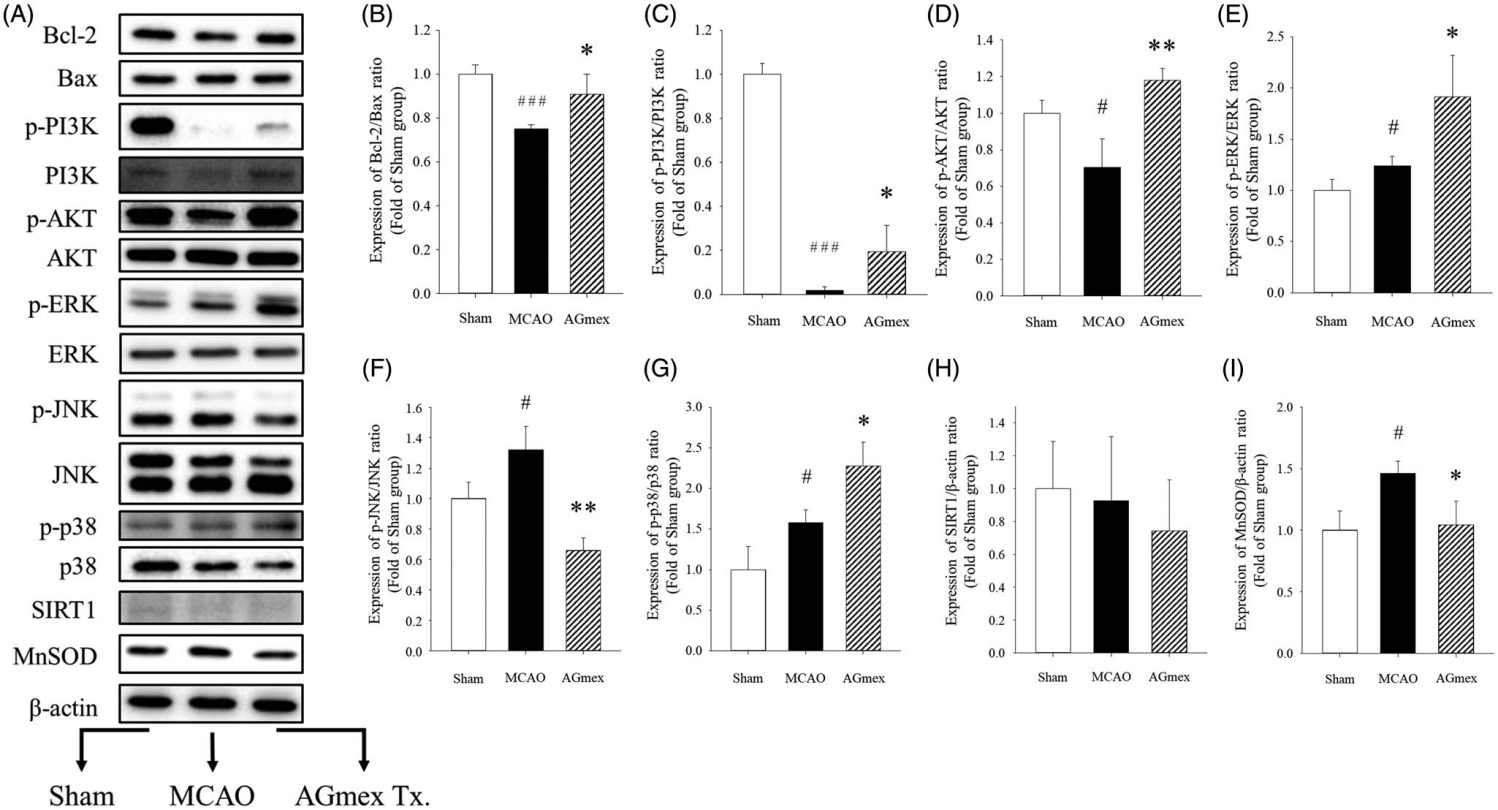
Effects of double pre-treatment (at 1 h and 24 h pre-MCAO) with AGmex at 1000 mg/kg on the proteins expressed in MCAO-induced mouse brains. **(**A) Representative western blot image of the expression of Bcl-2, Bax, p-PI3K, PI3K, p-AKT, AKT, p-ERK, ERK, p-JNK, JNK, p-p38, p38, SIRT1, MnSOD, and β-actin in MCAO-treated mouse brain. B-I, ratios of the expression levels of signalling proteins: **(**B) Bcl-2/Bax; C, p-PI3K/PI3K; D, p-AKT/AKT; E, p-ERK/ERK; F, p-JNK/JNK; G, p-p38/p38; H, SIRT1/β-actin; and I, MnSOD/β-actin. Results are presented as means ± SDs (*n* = 3). #*p* < 0.05, ###*p* < 0.001 vs. sham-operated controls; *p < 0.05, **p < 0.01 vs. the MCAO group.

Protein expression levels are shown in Figure 7(A). The Bcl-2 and Bax proteins participate in mitochondria-related apoptosis (Cao et al. 2001; Guo et al. 2016). The results (Figure 7(B)) showed that tMCAO induced neuronal apoptosis and that two consecutive administrations of AGmex at 1000 mg/kg bw prior to tMCAO protected the neurons from apoptosis.

The PI3K/AKT/mTOR signalling pathway regulates various cellular functions, including differentiation, proliferation, survival, and autophagy (Prasad et al. 2011; Guo et al. 2016). IHC staining showed that mTOR was overexpressed in the ipsilateral hemisphere in the tMCAO group (red arrows) (Figure 6(B)), and that this overexpression was lower in the AGmex 1000 mg/kg-pre-treated group. The p-PI3K/PI3K (Figure 7(C)) and p-AKT/AKT (Figure 7D) expression ratios were lower in the AGmex 1000 mg/kg-pre-treated group than in the tMCAO group, indicating that AGmex reduced the tMCAO-induced cellular stress.

MAPK signalling was activated in the tMCAO group (Figure 7(E,F,G)), and ERK signalling (a major signalling cassette of MAPK related to cell growth and differentiation (Plotnikov et al. 2011)) was activated in the AGmex 1000 mg/kg-pre-treated group (Figure 7E). JNK activates apoptotic pathways by upregulating pro-apoptotic genes (Chuang SM et al. 2000; Plotnikov et al. 2011); the tMCAO-induced increases in JNK expression were inhibited by AGmex (Figure 7(F)). The p38 signalling pathway regulates apoptosis by targeting members of the Bcl-2 protein family (Chuang SM et al. 2000; Chuang WL et al. 2016); its expression was upregulated in the tMCAO group and highly upregulated in the AGmex 1000 mg/kg group (Figure 7(G)).

Several researchers have reported that some herbal medicines and plant-derived compounds attenuate cerebral ischaemic injury in a SIRT1-dependent manner (Zhu et al. 2010; Zheng et al. 2018). However, in the present study, neither tMCAO nor AGmex had any noticeable effect on SIRT1 expression (Figure 7(H)).

MnSOD, which is specifically localized to the inner mitochondrial membrane, is related to antioxidant pathways (Kinoshita et al. 2013; Ambe et al. 2014; Lu et al. 2015) and protects neurons from oxidative stress (Garnier et al. 2001;Scorziello et al. 2007; Jung et al. 2009), suggesting that MnSOD may be targeted by herbal agents that attenuate ischaemic brain injury. We observed that tMCAO significantly increased the expression of MnSOD, and pre-treatment with AGmex twice at 1000 mg/kg significantly inhibited this increase (Figure 7(I)).

## Discussion

*Angelica gigas* and its constituents have been reported to have neuroprotective, anti-inflammatory, reactive gliosis-suppressing, anti-dementia, antifungal, antiplatelet, anticoagulation, antioxidant, and anticancer effects *in vivo* and *in vitro* (Lee YY et al. 2003; Yan et al. 2004; Kang et al. 2005; Choi IJ et al. 2009; Song et al. 2011; Yim et al. 2011; Yoon et al. 2011; Shin and Park 2014; Cho et al. 2015; Li L et al. 2015; Oh et al. 2015; Choi HS et al. 2016). Oh et al. (2015) reported that the hairy root extract of *A. gigas* had a neuroprotective effect in rat MCAO models. Shin and Park (2014) compared the efficacies of extracts of different parts of *A. gigas*. Song et al. (2011) reported the effect of intravenous injection of an extract of AGR. These studies are similar to our study in that they used a rodent MCAO model to investigate the effect of *A. gigas,* but different because they used rats and water extracts and did not measure rCBF to assess the activity of AGmex. Interestingly, Song et al. (2011) showed that intravenous treatment with AGR extract had neuroprotective effects, which indicated that AGR extracts might be therapeutically useful. In the present study, the most efficient dosage was found to be 1,000 mg/kg of AGmex administered at 1 and 24 h before tMCAO (Figure 3); thus, we used this dosage regime to investigate the neuroprotective effect of AGR.

AQP4 is important for maintaining brain water balance by regulating BBB integrity, and brain edoema is a major treatment target in ischaemic stroke (Badaut et al. 2014; Wang et al. 2014). We found that AGmex attenuated ischaemia-induced cerebral injury by regulating AQP4 protein expression (Figure 4). Furthermore, cresyl violet staining of neurons and H&E staining of the nuclei and cytoplasm revealed that AGmex pre-treatment before MCAO improved the neuronal density and morphology in cresyl violet- or H&E-stained brain sections as compared with those in the tMCAO controls (Figure 5); additionally, more apoptotic neurons were observed in the tMCAO group than in the AGmex 1000 mg/kg-pre-treated group (Figure 6(A)). Interestingly, the mTOR protein [a kinase and important regulator of cell growth, proliferation, and survival (Prasad et al. 2011; Guo et al. 2016)] was overexpressed in both the tMCAO and AGmex 1000 mg/kg-pre-treated groups, suggesting that tMCAO induced cell proliferation (Figure 6(B)). These results indicate that AGmex inhibited the tMCAO-induced apoptosis signalling, but did not influence MCAO-induced cell survival signalling.

Furthermore, PI3K/AKT signals were upregulated in the brains of AGmex 1000 mg/kg-pre-treated mice (Figure 7(B–D)). Considering that MAPK is an intracellular signalling molecule that promotes cell migration, proliferation, and differentiation, and that its relevant sub-molecules, namely ERK, JNK, and p38, are downstream of MAPK (Sun and Nan 2016), the expression of ERK, JNK, and p38 observed in this study (Figures 7(E–G)) indicated that AGmex treatment upregulated the MAPK pathway.

MnSOD appears to protect neurons against oxidative stress (Ambe et al. 2014; Lu et al. 2015) and is an important target for agents that impact ischaemic brain injury. In this study, tMCAO significantly increased MnSOD protein expression in the ischaemic ipsilateral hemisphere, and AGmex pre-treatment at 1000 mg/kg significantly inhibited this increase, suggesting that antioxidative signals were activated by tMCAO, and that the reduced level of cerebral damage elicited by AGmex pre-treatment suppressed MnSOD protein levels (Figure 7(I)).

We used ND as the positive control based on previous reports of its potential positive effect on ischaemic stroke (Zhao et al. 2018). However, ND had no positive effect on our animal model. Furthermore, the mean NDS of mice administered ND at 60 mg/kg bw post-MCAO was higher than that of the mice in the tMCAO group, suggesting that caution should be exercised when deciding whether to administer ND to patients with ischaemic brain diseases.

**Figure 8.**
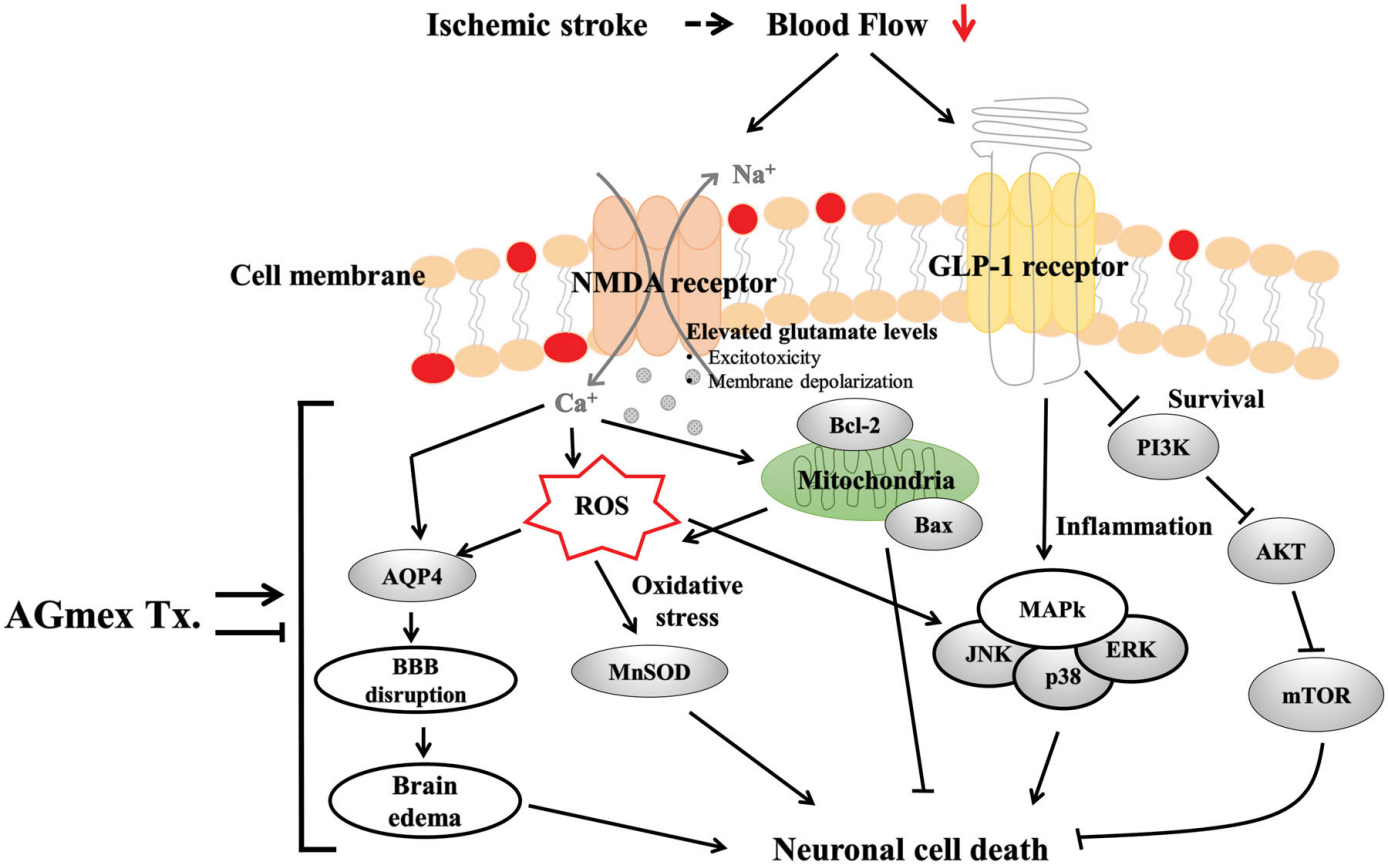
Schematic view of the suggested cerebral damage pathways and the neuroprotective mechanism of AGmex. AGmex ameliorated mitochondrial function and MAPK signalling pathways in the neurons exposed to ischaemia, and the neuroprotective effects of AGmex were found to mainly involve anti-edoema and anti-apoptotic pathways.

In summary, AGmex improved mitochondrial function via the Bcl-2/Bax, PI3K/AKT/mTOR, and MAPK signalling pathways in neurons exposed to ischaemia, suggesting that AGmex activates proteins involved in cell survival and inhibits those involved in cell death. The suggested mechanisms and pathways involved in the neuroprotective effects of AGmex are summarized in Figure 8.

*Angelica gigas* is a medicinal plant that is widely used in traditional Korean medicine, but not in Chinese medicine. This study confirmed that pre- and post-treatment with AGR, which is used in Korean medicine, exerts a neuroprotective effect in an ischaemia-induced brain infarction mouse model; in addition, some of the mechanisms involved in this effect were investigated. However, the treatment had no effect on changes in NDS. The reason may be the short behaviour observation period in our study. Thus, we plan to investigate whether NDS can improve with longer behaviour observation period, in our future study.

## Conclusions

Percentage neuronal cell death was reduced by AGmex orally administered at 1,000 mg/kg at 1 and 24 h before tMCAO induction as compared with that in the tMCAO controls; this protective effect appeared to involve anti-edoema and anti-apoptotic pathways, suggesting that AGmex attenuated the MCAO-induced BBB disruption and the upregulation of mitochondria-related cell survival signals. Thus, AGmex appears to protect neurons and inhibit apoptosis by tMCAO. This study provides preclinical evidence of the potential neuroprotective effects of AGR in the context of ischaemic stroke.
